# A Self-Standing Binder-Free Biomimetic Cathode Based on LMO/CNT Enhanced with Graphene and PANI for Aqueous Rechargeable Batteries

**DOI:** 10.3390/ijms23031457

**Published:** 2022-01-27

**Authors:** Constantin Bubulinca, Irina Sapurina, Natalia E. Kazantseva, Viera Pechancova, Petr Saha

**Affiliations:** Centre of Polymer Systems, Tomas Bata University in Zlin, Tr. T. Bati 5678, 760 01 Zlin, Czech Republic; sapurina@utb.cz (I.S.); kazantseva@utb.cz (N.E.K.); pechancova@utb.cz (V.P.); saha@utb.cz (P.S.)

**Keywords:** rechargeable Li-ion aqueous battery, conductive polymers, PANI core-shell coating, biomimetic electrode

## Abstract

The electrochemical parameters of a novel binder-free self-standing biomimetic cathode based on lithium manganese oxide (LMO) and carbon nanotubes (CNT) for rechargeable Lithium-ion aqueous batteries (ReLIAB) are improved using polyaniline (PANI) core-shell in situ polymerization and graphene (Gr). The fabricated cathode material exhibits the so-called “tectonic plate island bridge” biomimetic structure. This constitution is created by combining three components as shown by a SEM and a TEM analysis: the Gr substrates support an entangled matrix of conductive CNT which connect island of non-conductive inorganic material composed of LMO. The typical spinel structure of the LMO remains unchanged after modifying the basic structure with Gr and PANI due to a simplified hydrothermal method used for synthesis. The Gr and PANI core-shell coating improves the electric conductivity from 0.0025 S/cm up to 1 S/cm. The electrochemical performances of the LMO/CNT-Gr/PANI composite electrode are optimized up to 136 mA h g^−1^ compared to 111 mA h g^−1^ of the LMO/CNT. Besides that, the new electrode shows good cycling stability after 200 galvanostatic charging/discharging cycles, making this structure a future candidate for cathode materials for ReLIAB.

## 1. Introduction

In recent decades, new types of smart electronics, wearable devices, and rollup displays have appeared which require adaptation of the electrochemical features of the energy storage technology [1,2]. Currently, the market is led by Lithium-ion battery (LIBs) technologies in different structures, such as coin, pouch, prismatic, or cylindrical types, but it is still necessary to improve the main electrochemical parameters, especially power density. It is known that the cathodes can be fabricated by the classic method from a paste based on an active material, a conductive carbon material, a binder, and a solvent applied to a metal sheet. Electrodes of this type have low flexibility due to their rigid construction. However, this feature can be changed using a robust matrix based on carbon nanotubes (CNT) and graphene (Gr) networks for conductive and flexible binder-free electrodes [3,4,5,6]. The lithium manganese oxide (LMO) spinel type is one of the most popular active materials used due to its abundance, good electrochemical properties, low price and the option to facilitate the intercalation of Li+ ions [7]. However, LMO also has a few disadvantages, among them is the dissolution of Mn^3+^ and structural degradation caused by an electrochemical reaction connected to low electronic conductivity which definitely limits their application in high power LIBs [8,9,10]. The Jahn–Teller distortion of Mn^3+^ is also one of the reasons for capacity fade of LMO spinel cathode materials for LIBs [10]. In order to avoid Mn^3+^ dissolution many efforts have been made by scientists using different approaches, such as surface coating, changing electrolytes, and so on [10]. Rechargeable Lithium-ion aqueous battery (ReLIAB) technologies is an alternative to organic cells, although few researchers have explored this opportunity, it integrates this type of electrode into systems that use aqueous electrolyte. The most important advantages of ReLIAB are low production costs, a better level of safety, and also a higher ionic conductivity than organic secondary cells [3,4,11,12]. The main disadvantage is the limitations in energy densities. Moreover, a coating technology was considered as a possible solution for the issue of Mn^3+^ dissolution, thus numerous approaches using organic and inorganic substances have been developed [13]. It can be mentioned here that coatings for an LMO surface using metal oxides include Al_2_O_3_ [13,14] and ZrO_2_ [15,16], SiO_2_ [17], salts AlF_3_ [18] or AlPO_4_ [19], or MnO_2_ spinel [20] and conductive polymers such as polyaniline (PANI) and polypyrrole (PPy) [21] or doping using Ni-Co-Mn [22], Co-Mg-Ni [23] in order to protect against exposure to electrolytes.

Recent research findings show that a poly (ethylene glycol) (PEG), (CNT), lithium manganese oxide (CNT@PEG@LMO) composite prepared by the ultrasound-assisted wet-coating method show an initial discharge capacity of up to 123.8 mA h g^−1^ at 1.0 C with capacity retention of 95.2% after 100 cycles [24]. A hybrid nano-LMO-Gr-CNT material synthesized by a facile hydrothermal method demonstrates initial discharge capacity of 129 and 100 mA h g^−1^ at 0.2 and 5.0 C, and still above 93 mA h g^−1^, and capacity retention of over 82.1% after 600 cycles at 2.0 C [25]. Moreover, a 3D porous MnO@Gr/CNT anode composite was reported which exhibits an ultrahigh capacity of 1115 mA h g^−1^ at 0.2 A g^−1^ after 150 cycles and outstanding rate capacity of 306 mA h g^−1^ at 10.0 A g^−1^ [26].

Conductive polymers (PANI and PPy) attract the attention of scientists due to their special properties such protecting against corrosion, electrical conductivity; and are often used in secondary batteries and supercapacitors [21,27], microwave absorbers [28], and electromagnetic interference shielding materials [21] etc. PANI is one of the most explored conductive polymers due to its electrical conductivity and good mechanical properties (flexibility, stretching) [29] and thus was recently involved in studies focused on rechargeable batteries and supercapacitors [30].

Herein, we have undertaken a comparative study of binary and ternary self-standing composite materials prepared by an optimized binder-free technology presented in our previous work [31]. This work has resulted in an LMO/CNT binary “island–bridge” and an LMO/CNT-Gr ternary “tectonic plate–island bridge” biomimetic structure development. Multilayered graphene has been used as the “tectonic plate” substrate on which stands the islands of the LMO active material interconnected by a bridged network of multiwall CNTs. Thereafter, the LMO/CNT-Gr composite has been coated in situ with a thin PANI layer to form a core-shell structure. A comparative analysis of the obtained cathode materials shows the advantage of LMO/CNT-Gr/PANI in terms of physicochemical parameters, emphasizing its electrochemical potential and the possibility of integrating it into water hybrid storage batteries.

## 2. Materials and Methods

### 2.1. Materials

The sintered LMO with a spinel-type structure was purchased from MTI Co. Ltd., USA. where it was produced by heat treating (750 °C in air) a mixture of lithium salt (e.g., CH_3_COOLi × 2H_2_O) and manganese oxide.

The graphene was produced by a chemical vapor deposition of graphene on metallic substrates of copper wafers.

The polyaniline was obtained from an aqueous solution of the monomer (aniline 0.01 M + o-phenylenediamine 0.001 M) in 20 mL 1 M H_2_SO_4_ and ammonium persulfate 0.01 M in 10 mL 1 M H_2_SO_4_.

The multiwall carbon nanotubes (MWCNT), with a length of approximately 1.5 μm and a diameter of approximately 10 nm, were purchased from Nanocyl Company (Belgium), where they are produced by a chemical vapor deposition method.

A Triton X-100 or poly (ethylene glycol) p-isooctyl-phenyl ether from Sigma Aldrich, Merck Life Science spol, an affiliate of Merck KGaA (Darmstadt, Germany) was used as a surfactant for preparing the aqueous dispersions.

The polyvynilidene fluoride (PVDF) membranes, with a pore size of below 200 nm and a total volume of pores greater than 70%, were purchased from Millipore Corporation (USA).

### 2.2. Fabricating the Self-Standing Binder-Free Biomimetic Cathode

**Figure 1:**
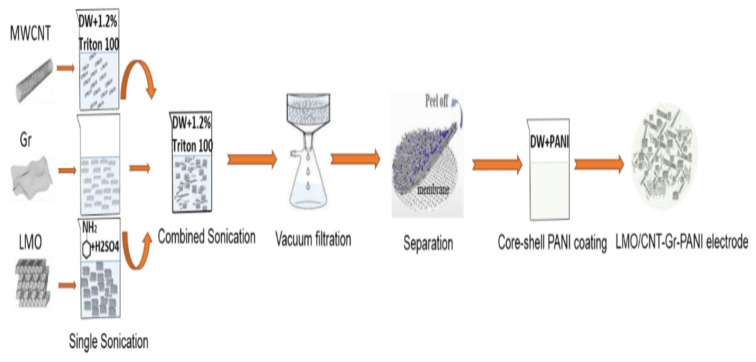
Schematic illustration of binder-free self-standing biomimetic composite material fabrication.

The method for fabricating the self-standing binder-free biomimetic cathode is described in Figure 1 and the following steps:

- Calculating the stoichiometric mass ratio of the electrode

The weight flow ratio of the materials required for the synthesis of the electrodes was calculated per 200 mg of the total mass: 9/1 LMO/CNT, 8.5/1–0.5 LMO/CNT-Gr and the same ratio for the LMO/CNT-Gr/PANI composite.

- Dispersion by ultrasonication

The materials used in powder form (LMO, CNT and Gr) were dispersed in approximately 50 mL of solution with Triton X-100 surfactant assisting for 30 min, maintaining the mixture at a constant temperature. Moreover, a 30 min ultrasonic treatment of LMO with CNT was carried out to form the LMO/CNT and the LMO, CNT and Gr to form the LMO/CNT-Gr.

- Vacuum filtration

The aqueous solution, using scattered particles of the materials, was separated by vacuum filtration with a Buchner funnel and a 2.5 cm diameter polymeric membrane until the composite electrode materials were deposited onto the substrate.

- Separation of the electrode from the filter

The composite materials formed a self-contained biomimetic structure which did not contain a binder. They were washed several times with demineralized water to remove the surfactant and then peeled-off from the substrate and dried in air.

- Thermal treatment

The thermal treatment of the electrode materials was undertaken in an oven at 110 °C under a controlled atmosphere for 4 h in order to remove residual water.

- Electrode coating by PANI

The active material of the biomimetic composite structure as well as the self-standing binder-free cathode were modified with polyaniline by in situ polymerization, i.e., during the synthesis of a conjugated polymer in the presence of a modified material. The LMO powder (0.5 g) was dispersed into an aqueous medium, then, into the suspension, with stirring, aqueous solutions of the monomer (aniline 0.01 M + o-phenylenediamine 0.001 M) in 20 mL 1 M H_2_SO_4_ and ammonium persulfate 0.01 M in 10 mL 1 M H_2_SO_4_ were added. The self-standing cathode was modified by a short exposure of the sample to a solution of a monomer (0.01 M aniline + 0.001 M o-phenylenediamine) in 20 mL of 1 M H_2_SO_4_ followed by the addition of a 0.01 M ammonium persulfate solution in 10 mL of 1 M H_2_SO_4_. Pure PANI, as a reference material, was obtained under the same conditions. The synthesis proceeded at 20 °C and finished after a few minutes with the formation of dark green PANI in the emeraldine conductive form. The precipitates of the powdery materials were decanted onto a filter, washed with water and ethanol, and dried in air. The self-standing cathode was rinsed off with water and dried.

### 2.3. Electrochemical Measurements

The electrochemical measurements of the binder-free self-standing composite were performed in an aqueous neutral electrolyte (1 M Li_2_SO_4_) using cyclic voltammetry (CV) at a scan rate of 0.1 mV s^−1^; the galvanostatic charge–discharge (GCD) using various current rates in a range of 0.3, 0.6, 1, 2 and 5 C; and the electrochemical impedance spectroscopy (EIS) in a frequency band from 0.1 to 50 kHz. The current rate was calculated according to LMO theoretical capacity (1 C = 148 mA h g^−1^). The electrochemical window potential used for measurements was between 0.4 and 1.2 V vs. Ag/AgCl for the electrodes which contained LMO/CNT and LMO/CNT-Gr, while for the samples which contained PANI it was between −0.3–1.2 V. The electrochemical measurements were performed in a three-electrode system with an Ag/AgCl reference electrode and a platinum counter electrode. The specific capacity was calculated with regard to the total mass (2 mg of active material) involved in the reaction of the self-standing binder-free biomimetic electrode.

### 2.4. Characterization

The morpho-structural properties of the materials were studied by a scanning electron microscope (SEM, FEI Nova NanoSEM450, Thermo Fisher Scientific) operating at 120 kV and transmission electron microscopy (TEM/HRTEM, JEOL JEM2100F). In addition, the SEM included an EDAX Energy Dispersive X-Ray Spectroscopy (EDS) system, which is used to perform qualitative analysis of material dispersion on the surface of a composite electrode. The frequency dependence (0.1–10 MHz) of alternative current (AC) conductivity were performed as frequency sweeps, in a temperature range from 0 to 50 °C, in 5° steps, with an amplitude of measuring voltage equal to 1 V, using an impedance analyzer (Novocontrol, Germany), on thin layer films of samples, in the form of 80 µm thick discs with a diameter of 15 mm. The crystal structure of the LMO was analyzed with the help of the X-ray diffraction technique (XRD, Rigaku MiniFlex 600, Japan) with Cu-Kα radiation, 0.1540560 nm wavelength and current of approximately 1 μA in a range of 3–90°. The X-ray photoelectron spectroscopy (XPS) measurements were performed using a photoelectron spectrometer K-Alpha XPS system (Thermo Fisher Scientific) with a monochromatic Al Kα source, in a range of 0–1300 eV for the survey. The assessment of the electrochemical performance was performed at room temperature using an Autolab PGSTAT-128 N potentiostat from Metrohm, Utrecht, Netherlands.

## 3. Results and Discussion

### 3.1. The Composition and Morphology of Electrodes

**Figure 2:**
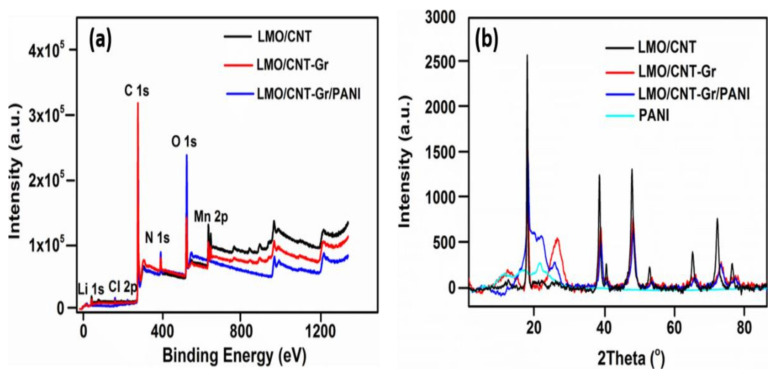
Survey XPS spectra of LMO/CNT, LMO/CNT-Gr, and LMO/CNT-Gr/PANI within a range of energy from 0 to 1350 eV (**a**) and XRD spectra of the PANI, CNT/PANI and LMO/PANI (**b**).

The composition of the surface chemical elements of the fabricated composite electrodes were investigated by an XPS and are shown in Figure 2a. Therefore, the LMO/CNT, LMO/CNT-Gr, and LMO/CNT-Gr/PANI samples were scanned in a survey with binding energy ranges from 0 to 1350 eV. The spectrum showed that the investigated samples contained following main elements: Li 1s, Cl 2p, O 1s, C 1s, Mn 2p, and N 1s suggesting that the composite material was formed with a different stoichiometric composition corresponding to peak intensity variation. The XRD spectra measured for the LMO/CNT, LMO/CNT-Gr, LMO/CNT-Gr/PANI, and PANI are presented in Figure 2b. The characteristic patterns of the PANI were found at 2θ ~ 23° and had a broad-shape characteristic for materials with an amorphous structure. Whereas, the peaks of the LMO/CNT, with the LMO in a 1:2 molar ratio Li:Mn situated at 2θ = 19, 38, 40, 48, 54, 64, 75 and 77° revealed the pattern of the spinel structure of the LMO with a cubic symmetry with a Fd3m space group corresponding to (111), (311), (222), (400), (331), (440), (533) and (622) planes (JCPDS card no. 35–0782) [14].

Increasing the amount of carbon material in the sample and adding the Gr resulted in two new peaks at 2θ~12 and 26°, corresponding to the (001) and (002) lattice plane [32]. The intercalation of the oxygen functional group in the graphite structure lead to an increase in the interlayer space, thus shifting the peak to 12°, which is characteristic of Gr [33]. The XRD spectra after the PANI coating of the LMO/CNT-Gr self-standing binder-free composite electrode was slightly changed at 23° with a broader peak due to the amorphous structure of the PANI. However, intensity of the peaks decreased once we added Gr and PANI into the basic structure by increasing the sonication time and reducing the particle size. The shift of the diffraction peaks at lower 2θ degree angles and the broadening of the diffraction peaks was associated with a decrease in the crystallite size during the sonication procedure. Thus, for larger crystallites (LMO pristine), the diffraction peaks were produced at the precise location of the Bragg angle due to coherent scattering within the structure. For smaller particles, the broadening of the diffraction peaks and a small deviation from the Bragg angle were observed [34].

**Figure 3:**
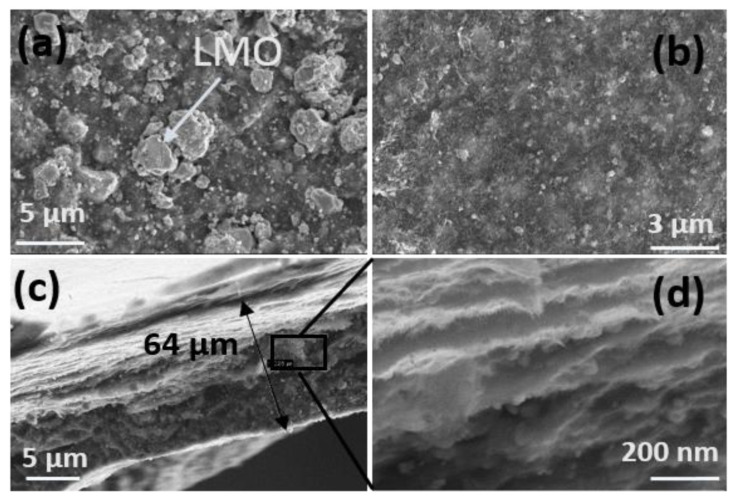
Morphology of self-standing binder-free biomimetic composite LMO/CNT and LMO/CNT-Gr electrode (**a**,**b**) and SEM image of the electrode section and the layered structure (**c**,**d**).

Figure 3 shows the morphology, i.e., thickness and layered construction, of the LMO/CNT and the LMO/CNT-Gr self-standing binder-free biomimetic electrodes by SEM images. On the surface of the electrode homogenously dispersed micron and nanosized particles of LMO (Figure 3a) were seen, interconnected by a support network of CNT visible at higher magnification (Figure 3b). 

The SEM cross-section image of the LMO/CNT-Gr composite cathode exhibits a thickness of approximately 64 µm (Figure 3c) which can be considered an optimal size for cathode materials as is described in [35]. According to the SEM images (Figure 3d), the geometry of the cross-section of the electrode consists of superimposed layers of a “tectonic–plate island–bridge” biomimetic-like structure. This arrangement provides fast kinetics and reduces the ion path length supported by a tunneling effect created by a CNT matrix.

**Figure 4:**
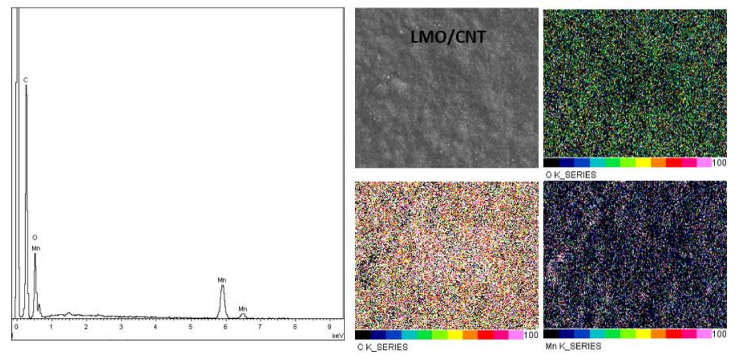
Elemental analysis of the LMO/CNT by EDAX.

In order to analyze the effectivity of the dispersion and the homogeneity of a particle in the basic structure of the LMO/CNT we performed an energy dispersive X-ray measurement (Figure 4). Ultrasonication in an aqueous solution by a surfactant created a uniform structure in which the elements were well-dispersed in concordance with the elemental mapping, as shown in Figure 4. Moreover, the EDAX spectra contains peaks corresponding to the CNT (C peak), O and Mn, the chemical elements contained in the LMO/CNT sample were without any contamination.

**Figure 5:**
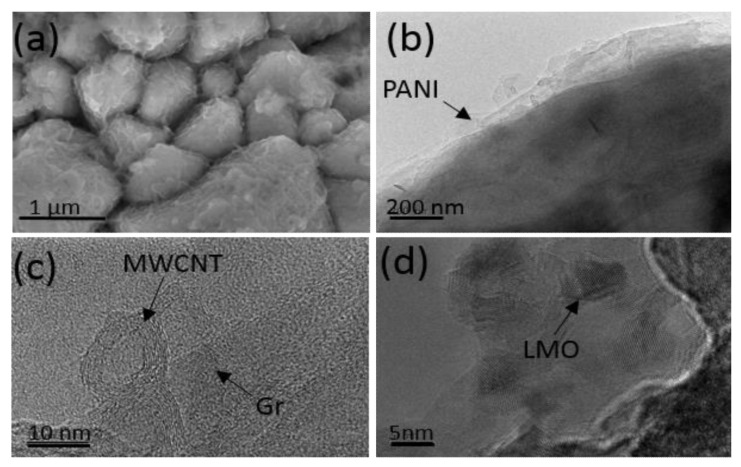
SEM image of the LMO/CNT-Gr/PANI composite (**a**), HRTEM image of core-shell coating of self-standing binder-free electrode by PANI (**b**), HRTEM of MWCNT and Gr (**c**), and HRTEM of LMO nanoparticles (**d**).

The morphological structure of the self-standing binder-free composite electrode based on LMO/CNT-Gr/PANI was investigated by SEM and HRTEM (Figure 5). The SEM image of the PANI core-shell coated composite is shown in Figure 5a and shows the active particle structure of the LMO with a different particle size, from hundreds of nanometers to several micrometers. Moreover, a semitransparent waved layer of the PANI coating the entire surface of the composite electrode can be seen on the top of the structure. The structure of the composite electrode was studied in more detail using a HRTEM (Figure 5b) with which it can be observed that the PANI layer has a variable thickness of several nanometers. Figure 5c shows the position of the graphene structure located between two multilayer carbon nanotubes which interconnect the LMO particles (Figure 5d) to create the so-called “tectonic plate–island bridge” biomimetic structure.

### 3.2. Conductivity Measurements

The binder-free self-standing biomimetic composite is multi-component, it includes the main energy-saving LMO material, carbon compounds, and a conducting polymer. The morphological study showed that the listed components were dispersed at nano-level and were uniformly distributed to create a homogenous composite electrode material. It is important to know how the components of a given system interact with one another. PANI composites with carbon compounds are well-studied [36,37]. It is shown that in the course of in situ polymerization, particles of carbon materials are covered with a hydrophilic polymer nanolayer and the electrical conductivity of the composite does not depend on the electrical conductivity of PANI as long as the PANI content does not exceed the carbon mass [38]. Unlike carbon materials, the interaction of PANI with LMO has hardly been studied. Thus, it was necessary to fill this gap, especially as the LMO is the main part of the electrode.

The bulk conductivity of the core-shell structured nanorods of the LMO@PANI was measured by the four point method and found to be 7.04 × 10^−5^ S/cm, 1.46 × 10^−3^ S/cm, and 7.89 × 10^−2^ S/cm for the LMO, the PANI, and the LMO@PANI, respectively [39]. Moreover, the frequency dependence of the AC conductivity (σAC) of the LMO, the PANI, and their composite at room temperature were evaluated; the AC conductivity at all applied frequencies increased in the order: LMO@PANI (5.3 × 10^−2^ S/cm) > PANI (8.1 × 10^−4^ S/cm) > LMO (6.06 × 10^−5^ S/cm) [39]. Thus the composite material LMO@PANI demonstrates an electrical conductivity that is orders of magnitude higher than the conductivity of the initial components.

A similar phenomenon has already been observed for composites of electrically-conducting polymers. In situ obtained composites PANI/Fe_3_O_4_ [40] and PPy/MoS_2_ [41], in which the polymer nanolayer is deposited on the particles of the inorganic material, also showed significantly higher electrical conductivity than their constituent components. In our opinion, this phenomenon is explained by a charge transfer between two semiconductors, i.e., the inorganic material and the conducting polymer. On contact, some of the electrons are transferred from the polymer nano-layer to the inorganic material. From a chemical point of view, the polymer is oxidized, the number of charge carriers—positive polarons—increases in it, and the electrical conductivity of the polymer layer increases. Indeed, manganese dioxide has a higher oxidation potential than PANI and is able to oxidize the polymer [42] and thereby increase the electrical conductivity of the particle encapsulating layer (Figure 5a), which forms the percolation network in the LMO@PANI.

**Figure 6:**
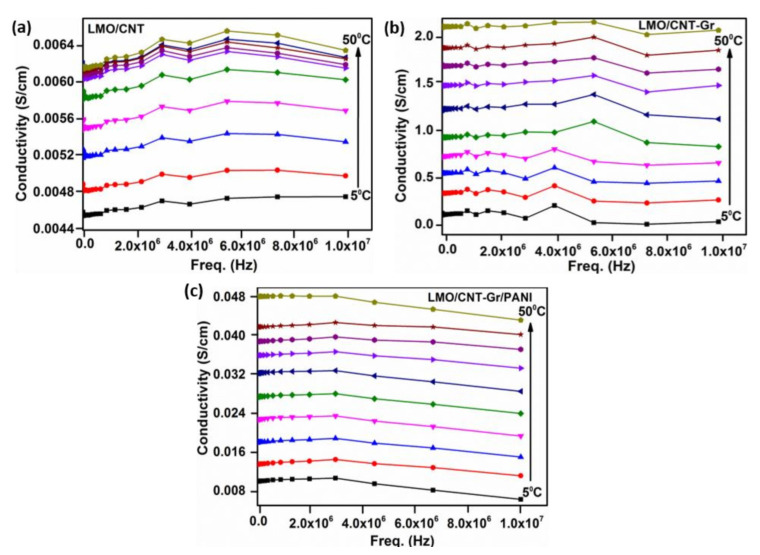
Electrical conductivity of LMO/CNT (**a**), LMO/CNT-Gr (**b**) and LMO/CNT-Gr/PANI (**c**) within a wide range of frequencies (0 to 10^7^ Hz).

The frequency dependence of the AC conductivity (σ*_AC_*) in the temperature range of 5 °C to 50 °C is shown in Figure 6. As shown in the figure, the electrical conductivity changes insignificantly with frequency but markedly with temperature. Besides which, the LMO/CNT and the LMO/CNT-Gr samples demonstrate a similar dependence of conductivity on the frequency number and temperature, i.e., σ*_AC_* increases with a temperature rise of up to 8MHz and then decreases, while the conductivity of the LMO/CNT-Gr/PANI decreases with an increasing frequency. This phenomenon can occur due to the hydrophilization of electrodes during preparation in the aqueous dispersion process [43], and the presence of hydrophilic PANI [36]. The electrical conductivity for self-standing binder-free biomimetic electrodes with various chemical compositions in a temperature range of 5 to 50 °C is given in Figure 6. There we comment on the increase in AC conductivity with temperature for entire ranges of temperatures. The LMO/CNT exhibits the lowest value of approximately 0.028 S/cm at room temperature compared with almost 1 S/cm for the LMO/CNT-Gr and ~0.06 S/cm for the LMO/CNT-Gr/PANI. The semiconductor materials exhibit similar behavior.

The increase in electrical conductivity can be explained by an increase in the amount of conductive material in the main composite structure (LMO/CNT), as well as by the arrangement of the components which provide high conductivity paths in a “tectonic plate–island bridge” biomimetic structure (Figure 3b and Figure 5c).

### 3.3. Electrochemical Characterization

**Figure 7:**
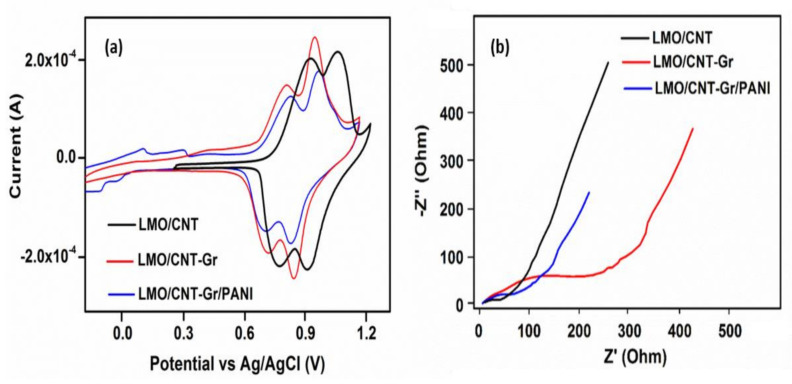
Cyclic voltammetry (**a**) and electrochemical impedance spectra (**b**) of LMO/CNT, LMO/CNT-Gr and LMO/CNT-Gr/PANI at a scan rate of 2 mV s^−1^ over the frequency range of 0.01–50 kHz after 5 GCD cycles.

The electrochemical experiments were performed in an aqueous neutral electrolyte (1 M Li_2_SO_4_) and an electrochemical window of approximately 1.5 V, from−0.3 V to 1.2 V in order to avoid critical potential of water decomposition at 1.23 V. The cyclic voltammetry tests were measured at 2 mV s^−1^ scan rate and the results are presented in Figure 7a. The first cycle of the CV curve for the LMO/CNT describes two reduction/oxidation peaks that are almost symmetrical on both the cathodic (0.85 and 0.97 V) and the anodic (0.77 and 0.92 V) sides of the redox reactions, while the LMO/CNT-Gr feature is slightly shifted to the low potentials and the peaks become sharper. More specifically, the physicochemical process involved is correlated with the redox-type reactions of Mn^3+^↔Mn^4+^ [44] along with the ion-exchange type during shifts in a spinel structure [1]. The specific CV curves of the LMO/CNT-Gr/PANI show two sets of distinct redox activity as indicated by two pairs of anodic and cathodic current peaks. The first set of the redox couple which appears between −0.2 and 0.25 V vs. silver/silver chloride reference electrode (Ag/AgCl) is associated with the conversion of the fully reduced leucoemeraldine base to the partially oxidized emeraldine [45], and the second set of the redox current peaks occurring between 0.1 and 0.35 V vs. Ag/AgCl correspond to the conversion of emeraldine to the fully oxidized pernigraniline form [45]. The PANI content in the biomimetic composite electrode material is small and therefore the redox contribution is also small.

Modifying the structure, position, and sharpness of the peaks on the CV curves indicates that the kinetic for ion transfer during the cycling process was improved by adding the Gr and PANI into the basic structure of the self-standing binder-free composite material, thus revealing the intensification of the Faradaic reactions.

The Nyquist plots related to the kinetics transfer and the resistance fluctuation during the ionic transfer of the investigated self-standing binder-free electrodes are shown in Figure 7b. The impedance spectra measurement reveals a semicircle in the high-frequency range and a quasi-linear part in the low-frequency range. The presence of a semicircle is due to the resistance of the electrode to charge transport. The diameter of the semicircle is different, the highest resistance at the electrode/electrolyte interface belongs to the LMO/CNT-Gr, which can be explained by the hydrophobic properties of the carbon materials present and involved in the activation process which appears at the start of the electrochemical cycling. The corresponding impedance at high frequencies shows that the resistance for the LMO/CNT-Gr/PANI during the charge transfer from the electrode to the electrolyte exhibit the smallest value, thus it can be concluded that introducing PANI into the composite electrode causes an increase of Li+ insertion/extraction, which leads to improving the electrochemical performance [46]. The quasi-linear part at a low frequency is known as the Warburg element and appears during the Li-ion diffusion through the electrode material. Decreasing the angle and the length of the quasi-linear Warburg elements indicates increasing Li+ diffusion and charge transport rate in the self-standing binder-free biomimetic composite. Thus, the LMO/CNT-Gr/PANI electrode exhibits an improved Li-ion diffusion compared to the LMO/CNT and the LMO/CNT-Gr.

**Figure 8:**
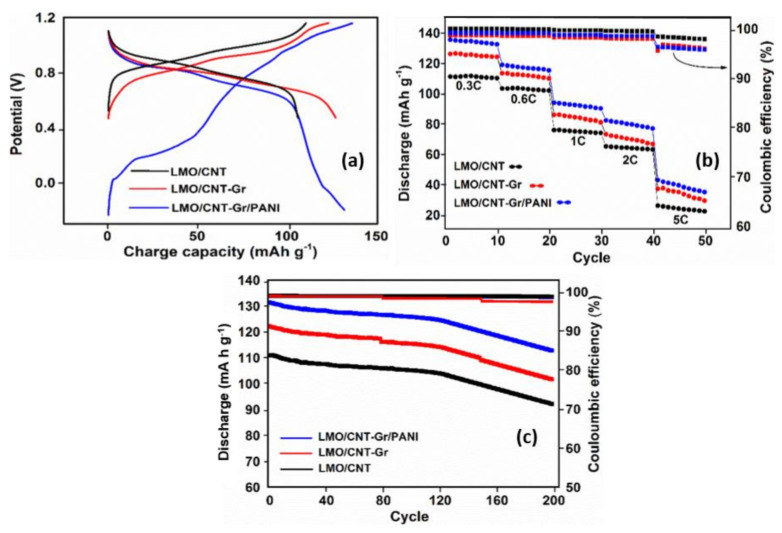
Galvanostatic charge/discharge curve of the first cycle at a scan rate of 0.15 mA (**a**), rate capability at 0.3, 0.6, 0.8, 1, 2 and 5 C (**b**) and cycling performances at 0.6 C for a series of 200 cycles (**c**) of self-standing binder-free LMO/CNT, LMO/CNT-Gr, LMO/CNT-Gr/PANI composite materials.

The specific capacity of the composite electrode based on the LMO/CNT was measured by the galvanostatic charge–discharge curves. The trend of the first charge–discharge curve (Figure 8a) at 0.3 C (0.15 mA) was plotted in a window potential from −0.3–1.2 V for the sample which contained PANI, and from 0.4–1.2 V for the LMO/CNT and the LMO/CNT-Gr composite electrodes. The results obtained show the presence of two plateaus and two semi plateaus for the composite electrode with PANI, which correspond with those identified on the CV curves. The litiathion/delithiation behavior identified on the LMO/CNT and the LMO/CNT-PANI curves exhibits only two plateaus corresponding to the redox reaction with the insertion/extraction of Li-ion in/from the anode to the cathode materials during the charge flow by the external circuit. Moreover, the specific charge capacity of the self-standing binder-free electrode based on LMO/CNT, LMO/CNT-Gr, and LMO/CNT-Gr/PANI after the first galvanostatic charge/discharge process was approximately 112/110, 126/127 and 135/133 mA h g^−1^, respectively.

The stability and ionic reversibility of the self-standing binder-free biomimetic composite electrodes was assessed by five series of ten charge/discharge cycles at different current rates: 0.3, 0.6, 1, 2 and 5 C. The results are shown in Figure 8b. A higher specific discharge capacity of approximately 136 mA h g^−1^ was obtained at 0.3 C for the composite electrode which contained PANI, while only 129 and 111 mA h g^−1^ were obtained for the LMO/CNT and the LMO/CNT-Gr samples. The GCD values were in agreement with the AC conductivity, the CV, and the EIS analysis measurements revealing that Gr and PANI improve the overall electrochemical properties due to an increase in electrical conductivity, a shortening of the kinetics path protecting against Mn dissolution and Jahn–Teller distortion and the individual synergy of the materials. It must be mentioned that even after the 5 C rate, the GCD curve still exhibits 47, 40 and 25 mA h g^−1^ for the LMO/CNT-Gr/PANI, LMO/CNT-Gr, and the LMO/CNT. At the same time, except for the 5 C rate, the variation of the Coulombic efficiency is rather low during the charging/discharging cycles, this demonstrates good electrochemical stability of the self-standing binder-free biomimetic composite material structure (Figure 8b). To prove the cycling stability of the composite electrodes based on LMO/CNT, the discharge capacity for 200 cycles at a 0.3 C rate and the corresponding Coulombic efficiency were analyzed (Figure 8c). According to the graph, all three cathode materials show a good electrochemical performance, with a small variation in discharge capacity up to the 120th GCD cycle, followed by an increasing loss of specific capacity, but with a retained specific discharge capacity of approximately 115 mA h g^−1^ after 200 cycles for the composite electrode which contained the PANI core-shell coating, thus a potential electrode for environmentally-friendly batteries can be made from this material. The Coulombic efficiency is nearly 100% for up to 160 cycles and around 97% at the end of cycling stability tests which shows good efficiency of the insertion/extraction of the Li-ion during the charging/discharging process. Therefore, the process of lithiation/delithiation occurs without a significant change in the structure of the binder-free self-standing cathode materials.

## 4. Conclusions

In this work, we have investigated the effect of a polyaniline coating forming a core-shell structure on a binder-free graphene-reinforced biomimetic composite electrode based on LMO/CNT. The composite materials were fabricated using a simplified hydrothermal method, resulting in three different composite electrode materials: LMO/CNT, LMO/CNT-Gr, and LMO/CNT-Gr/PANI. The effect of Gr and PANI, as assessed by the dielectric spectra, showed an important improvement in the electrical conductivity from 0.005 to 0.025 and 1 S/cm^2^, respectively. Graphene in conjunction with LMO and CNT creates a so called “tectonic plate–bridge island” structure which facilitates a better ionic kinetic due to shortening and tunneling the electrode/electrolyte transfer path. Moreover, the PANI core-shell coating of this structure by in situ polymerization creates a thin shield against Mn dissolution and a lower Jahn–Teller effect by decreasing the charge transfer resistance, thus improving the electrical conductivity and increasing the specific capacity due to an extended potential window. The electrochemical measurements reveal that the composite electrode with the core-shell PANI coating exhibits the best specific discharge capacity of approximately 136 mA h g^−1^ compared to 111 mA h g^−1^ of the LMO/CNT and a good cycling stability up to 200 GCD cycles, thus making this structure a candidate for environmentally-friendly cathode materials for rechargeable aqueous batteries.

## Data Availability

The data presented in this study are available on request from the corresponding author.
